# Decision-Making Capacity to Refuse Treatment at the End of Life: The Need for Recognizing Real-World Practices

**DOI:** 10.3390/clinpract12050079

**Published:** 2022-09-22

**Authors:** Akira Akabayashi, Eisuke Nakazawa, Hiroyasu Ino

**Affiliations:** 1 Department of Biomedical Ethics, Faculty of Medicine, University of Tokyo, 7-3-1 Hongo, Bunkyo-ku, Tokyo 113-0033, Japan; 2 Division of Medical Ethics, School of Medicine, New York University, 227 East 30th Street, New York, NY 10016, USA

**Keywords:** decision-making capacity, patient–doctor relationship, clinical ethics, end of life, trust

## Abstract

End-of-life decision making is a troublesome ethical dilemma. These decisions should be made in trustful patient–doctor relationships. We aimed to propose a balanced approach when discussing this complex issue. We categorized the research into four approaches and suggest that a multidisciplinary approach may be appropriate. We also analyzed the pitfalls of the multidisciplinary approach. Our conclusion is two-fold. First, discussions in this field should be based on real-world practice. If this is not the case, the proposal may be armchair theory, which is not effective in a clinical setting. Second, interdisciplinary researchers should not stick to their position too firmly and should listen to others. Otherwise, proposals made will be paternalistic or philosophically biased. Therefore, when philosophical collaboration is applied to the topic of clinical bioethics, it is necessary to thoroughly examine different positions and carry out careful discussions with consideration for medical care settings. Researchers must also understand what is needed for a trustful patient–doctor relationship. By making such efforts, clinical bioethics will contribute to the wellbeing of patients.

## 1. Introduction

End-of-life decision making is a troublesome ethical dilemma. These decisions should be made in trustful patient–doctor relationships. Numerous articles were found in PubMed using the keywords “decision making capacity”, “treatment refusal”, and “end of life”. The result was the same using other databases such as Philosophers Index. This short communication essay will not review all of these papers, but rather aims to propose a balanced approach when discussing this complex issue. We will focus on one paper as an example. When we searched the database using the above keywords, we found four categories of papers: (1) written mainly from the perspective of ethicists or philosophers [1,2,3]; (2) written mainly from a clinical perspective [4,5,6,7,8]; (3) written mainly from the perspectives of other fields such as law [9,10,11]; (4) written mainly by interdisciplinary expert authors such as clinicians and ethicists [12,13,14,15,16]. All categories have merits and demerits. It is evident to readers that papers in Category 1 will be logically robust but tend to focus only on theoretical reasoning; Category 2 papers will provide a lot of clinical information but may not be logical enough; Category 3 papers will be very informative, say, from legal perspectives, but that is their only scope. Accordingly, it seems that it is better to take the Category 4 approach, namely, papers written by both ethicists and clinicians working together. However, there are serious pitfalls in the Category 4 approach. 

## 2. Pitfalls of the Category 4 Approach

We will draw on one paper that took the Category 4 approach as an example [17]. The purpose is not to single out the authors for criticism, but to illustrate the risks when researchers take the Category 4 approach.

The authors criticize the “standard strict comparative” decision-making capacity (DMC) model and propose two new DMC approaches in the U.S. context. Namely, the “burden-based” DMC approach and the “goal-based” DMC approach. They first present the case of a 77-year-old female.

### 2.1. A Case

*Mrs. P was a 77-year-old female who had experienced a difficult medical course in recent months, including complications from chronic kidney disease, COVID pneumonia, dementia, meningioma, and a middle cerebral artery aneurysm. She was admitted to the hospital from her nursing home with an altered mental status, acute kidney injury, and abnormal labs following five days of refusing to eat or take medications, and she was repeatedly stating that she “just wants to die”. The patient was likely to survive to discharge with regular dialysis, medication, nutrition, and hydration. However, Mrs. P strongly refused any treatment, stating “I don’t want to be tied up here any longer; I don’t want any more procedures or tubes in me. I just want to go home”. The attending physician and a consulting psychiatrist determined that Mrs. P lacked DMC because she was not able to demonstrate a sufficient understanding of her treatment options or to reason. In their conclusion, the authors stated: “One goal of this paper was to provide a philosophical defense of real-world practices that are inconsistent with standard guidance, but which seem to be through the risks and benefits of those treatment options”. Following generally accepted views about the sliding-scale nature of DMC, they required Mrs. P’s capacities to reach a high threshold to determine that she possessed DMC, since she would likely die if she were allowed to refuse treatment. The attending physician stated that the patient clearly lacked DMC because her refusal would lead to her death, and because “she can’t even tell me the reason for the Quinton catheter in her chest”. The patient’s daughter was acting as the surrogate decision maker because Mrs. P had no partner or other children. The daughter was often present at the hospital and demonstrated consistent concern for Mrs. P’s wellbeing and a good understanding of the recommendations that physicians made about her mother’s care. Mrs. P’s daughter consented to all medications.*

[17], p. 2.

They then introduced the two new approaches. The authors defined the “burden-based” DMC approach as follows:

*“Some patients can make informed decisions to refuse possible medical interventions if they have an overriding objection to the burdens of those interventions, even if they are not capable of more complex comparative judgments regarding the nature and potential risks and benefits of the interventions they refuse. Such patients object to at least one burden associated with each of the proposed interventions, and they object to this burden so strongly that no reasonably foreseeable benefits of potential interventions could compensate for such a burden. That is, these patients recognize some overriding side constraints on their treatment decisions”.*

[17], p. 3.

The authors described a “goal-based” DMC approach as follows:

*“A second novel kind of DMC for refusal involves patients who are committed to the goal of non-treatment or to other goals that are inconsistent with possible medical interventions (e.g., going home). Some patients, for example, may want their disease to follow its natural progression. They may accept comfort measures, but they do not want to further prolong the course of their disease; they may have the goal of dying, or at least of no longer treating the disease. Importantly, a patient can make a goals-based refusal decision without understanding the details of their diagnosis and the nature and probable outcomes of possible treatments. It is enough that they know that they are being presented with possible medical interventions that interfere with their overriding goal”.*

[17] (pp. 4–5).

The authors stated that “one goal of this paper was to provide a philosophical defense of real-world practices that are inconsistent with standard guidance, but which seem to be morally justified”. We largely agree with the overall stance of this work, but the content of the paper did not fulfil the ideal, as it was faulty in the following two respects: dialysis and the placement of a nasogastric (NG) feeding tube. Because Mrs. P continued to object to these interventions, both the NG tube and dialysis required sedation and restraints. Mrs. P subsequently dislodged the NG tube. The physicians offered to place a percutaneous endoscopic gastronomy (PEG) tube, and the daughter consented. However, Mrs. P continued to refuse treatment, and the ethics consultation team was called to assist in determining how to proceed. 

The first author seemed to be a philosopher, and clinicians were also included, indicating that this was a Category 4 approach. Although the authors asserted a defense of real-world practices, it seemed that they did not have an enough understanding of real-world practices and psychology. Accordingly, they did not have an argument that would be valid in real-world practices. We will demonstrate this by analyzing potential risks in their approach.

### 2.2. What Is the Dominant Version of Decision-Making Capacity

Buchanan and Brock presented a framework and distinguished between three types of capacity standards: a minimal standard, an outcome standard, and a process standard [2]. The authors stated, citing Grisso and Appelbaum [18], that *"the dominant version of the DMC that has been embraced in major guidance documents for clinical ethics is a process standard."* How could the authors say that process standards are dominant? Yes, Applebaum’s claim was that the dominant version is a process standard, but does everyone in the real world have the same idea? Further, even individuals with moderate mental disabilities are considered competent enough to decide whether or not to treat their cavities.

The case of Mrs. P. was problematic. According to the standard (process) kind of DMC, Mrs. P. needed to have known what a “Quinton catheter” is. It was not strange that she did not know, because it is an uncommon term. We wonder why an elderly and seriously ill patient without any medical knowledge would be considered incapable of making a decision if she did not know about Quinton catheters. 

The first risk was that the philosopher’s own stance may have induced the direction of the whole approach even though there were clinicians involved in the team. In this case, there was an emphasis on individualistic autonomy.

There are many criticisms of individualistic autonomy and other types of autonomy. We believe the “relational autonomy” approach is a better fit for end-of-life discussions because it involves family, friends, healthcare professionals, and others.

#### 2.2.1. Burden-Based Refusal

A burden-based refusal should be based on at least one intolerable burden. The authors further stated:

*“They (Patients) do not need to know what the nature, benefits, and risks of various proposed surgeries are, or how a PEG tube differs from a nasogastric or jejunostomy tube, in order for them to have the capacity to make informed refusals of these options”.*

[17], p. 3.

We believe this statement is troubling because it is the opposite of the Quinton catheter issue; medical professionals cannot say that the differences between the purposes of a PEG and a nasogastric tube are unimportant. Moreover, the authors used speculative expressions:

*“It appears as if [there is a] good indication that there may be overriding burdens associated with possible interventions that she rejects”.*

[17], p. 4.

It might be said that the authors were not familiar with real-world situations and human psychology. Furthermore, the major philosophical flaw here was that the key word *burden* was not defined in a philosophically robust manner. How does this differ from *suffering*? Is it possible to treat the burden of moving house and that of a respirator at the same level? The comparison and delineation of quantitative and qualitative burden and suffering are indeed difficult, but in the real world, these comparisons and delineations are what bother medical practitioners the most—and are a point of contention in various court cases. It is true that Mrs. P. refused treatment, but what did she perceive as the burden? Was it the repeated insertion attempts of the IV needle or the discomfort around the nose? It may have been the fact that the familiar environment of her nursing home (e.g., her favorite chair and her favorite snacks) was not present in the hospital. Thus, the medical staff is required to identify these burdens and address them on a case-by-case basis.

#### 2.2.2. Goals-Based Refusal

The authors also stated the following:

*“Importantly, a patient can make a goals-based refusal decision without understanding the details of their diagnosis and the nature and probable outcomes of possible treatments. It is enough that they know that they are being presented with possible medical interventions that interfere with their overriding goal”.*

[17], p. 5.

If medical professionals are asked to approve a refusal of treatment even though the patient does not understand the diagnosis or prognosis, they will be in trouble. Is there no legal problem here? In addition, when a patient refuses certain treatment, it is necessary to deeply understand the background to their statements. Medical professionals are there for their patients in this way, and it is impossible to capture the nuances of asimple declarations of intent. Mrs. P. said, “I just want to go home,” which may not have been based on the refusal of treatment itself, but on a strong desire to return to her familiar nursing home. If this was her goal, switching from inpatient treatment to nursing home visitation would allow her to interact with familiar residents and feel secure. In other words, since a patient’s goal is dependent on their situation, considering only a patient’s refusal may overlook the patient’s true goal.

In addition, the authors stated the following:

*“The healthcare team should attempt to confirm that Mrs. P has consistently identified an overriding goal as the reason for her refusal, that her refusal is not the result of a delusion or mental illness, that she is not being coerced by others, that she understands (in a general sense) the gravity of the consequences that could result from her refusal”.*

[17], p. 4.

The medical staff needed to confirm that it was not a mental disorder, that there was no coercion, and that the refusal of treatment was serious. This is different from the previous burden-based model; specifically, this makes it seem that burden-based and goal-based patients with very different conditions have what would be considered DMC. A committed celibate in their paper exemplified the goal-based argument. It is not acceptable for medical professionals to equate DMC, a life-or-death treatment refusal, with a decision about celibacy or the burden of moving. Normal life burdens and goals are completely different from those at the end of life. Again, there was no robust philosophical definition of a goal. In more philosophical terms, what is the difference between a “desire” and a “goal”?

Moreover, goal-based refusal is a position that would allow voluntary euthanasia. The definition of “active” was out of the scope of this paper, since they were not, e.g., administering muscle relaxants, but if the patient was not taking food or water and refusing a PEG or a nanotube (natural death), they were not helping. Applying this idea to daily life, must a person who is about to hang themselves not be helped because of goal-based DMC? In this regard, this is a slippery slope argument.

Furthermore, as in the Baby K case [19], a mother with comparative DMC refused to provide comfort care to a newborn without a brain, and the infant lived for three years. Such examples are acceptable from a goal-based refusal perspective, but we cannot intuitively or perhaps philosophically accept them.

The second but most important risk was the following: discussions should be based on real-world practice in order to truly create proposals that will benefit patients. Recognizing real-world practice is, we argue, essential for the benefit and wellbeing of patients. Simply adding a philosopher or a clinician to a research team does not suffice. Those who really know real-world practice and human psychology are urgently needed in research of this kind.

## 3. Conclusions

Our conclusion is two-fold. First, discussions in this field should be based on real-world practice. If this is not the case, proposals may be armchair theory, which is not effective in clinical settings. Second, interdisciplinary researchers should not stick to their position too firmly and should listen to others. If this is not the case, proposals made will be paternalistic or philosophically biased. Therefore, when philosophical collaboration is applied to the topic of clinical bioethics, it is necessary to thoroughly examine different positions and carry out careful discussions with consideration for medical care settings. The researchers must also understand what is needed for trustful patient–doctor relationships. By making such efforts, clinical bioethics will contribute to the wellbeing of patients. 

## Data Availability

Not applicable.
